# Human Alpha-1 Antitrypsin Suppresses Melanoma Growth by Promoting Tumor Differentiation and CD8^+^ T-Cell-Mediated Immunity

**DOI:** 10.3390/biom16010122

**Published:** 2026-01-12

**Authors:** Takeshi Yamauchi, Yuchun Luo, Dinoop Ravindran Menon, Kasey Couts, Sana Khan, Aanchal Goel, Charles A. Dinarello, Zili Zhai, Mayumi Fujita

**Affiliations:** 1 Department of Dermatology, University of California San Diego, La Jolla, CA 92037, USA; tayamauchi@health.ucsd.edu (T.Y.); sak088@health.ucsd.edu (S.K.); aagoel@health.ucsd.edu (A.G.); 2 Department of Dermatology, University of Colorado Anschutz Medical Campus, Aurora, CO 80045, USA; yuchun.luo@csuglobal.edu (Y.L.); dinoop.ravindranmenon@cuanschutz.edu (D.R.M.); kasey.couts@cuanschutz.edu (K.C.); zili.zhai@cuanschutz.edu (Z.Z.); 3 Department of Medicine, University of Colorado Anschutz Medical Campus, Aurora, CO 80045, USA; charles.dinarello@cuanschutz.edu; 4 Department of Veterans Affairs Medical Center, VA Eastern Colorado Health Care System, Aurora, CO 80045, USA; 5 Department of Immunology and Microbiology, University of Colorado Anschutz Medical Campus, Aurora, CO 80045, USA

**Keywords:** alpha-1 antitrypsin, melanoma, melanin pigmentation, tumor microenvironment, immunity

## Abstract

Alpha-1 antitrypsin (AAT) is a serine protease inhibitor with potent anti-inflammatory and immunomodulatory properties, but its role in cancer is context-dependent across tumor types. We integrated transcriptomic analyses of human melanoma cohorts, in vivo studies using AAT-transgenic (hAAT-TG) mice, and in vitro assays in murine and human melanoma cells to define the biological functions of AAT in melanoma. *SERPINA1* expression increased progressively from normal skin to nevi and metastatic melanoma, yet higher intratumoral levels correlated with improved overall survival in metastatic disease. In hAAT-TG mice, melanoma growth was markedly inhibited compared with wild-type controls, and the inhibitory effect required CD8^+^ T cells and was enhanced by CD4^+^ T-cell depletion, demonstrating that AAT promotes cytotoxic T-cell activity while attenuating regulatory T-cell suppression. Histologic analysis showed heavily pigmented tumors in hAAT-TG mice. In vitro, hAAT upregulated melanocytic differentiation markers (MITF, TYR, PMEL, MART-1) and increased melanin production in murine and human melanoma lines, suggesting enhanced tumor immunogenicity. In conclusion, hAAT exerts antitumor effects in melanoma indirectly by reprogramming the tumor microenvironment toward differentiation and immune activation. These findings highlight a previously unrecognized role for AAT as a dual immunoregulatory and differentiation-promoting factor and support AAT as a potential immunoregulatory adjuvant in melanoma.

## 1. Introduction

Tumor-mediated inflammation is one of the hallmarks of cancer [1], promoting genetic instability, abnormal cell proliferation, angiogenesis, and immune evasion [2,3]. We and others have shown that inflammatory pathways within the tumor microenvironment (TME) contribute to melanoma progression, drug resistance, and immune suppression [4,5,6], underscoring the importance of understanding inflammation-driven mechanisms that could be therapeutically targeted.

Alpha-1 antitrypsin (AAT), a serine protease inhibitor primarily produced by the liver, exhibits anti-inflammatory, anti-angiogenic, and immunomodulatory activities [7]. Within the TME, AAT modulates multiple molecular and cellular processes, including inhibition of endothelial and leukocyte chemotaxis [8], NF-κB signaling [9], inflammasome/caspase-1 activation [7], and proinflammatory cytokine production [10]. AAT has also been reported to enhance M1 macrophage polarization and cytotoxic T cell killing [11]. Collectively, these effects suggest a potential tumor-suppressive and immune modulating role for AAT.

Extensive literature demonstrates that AAT exerts highly context-dependent effects across multiple cancer types. Elevated circulating AAT has been reported in lung, breast, prostate, colon, liver, biliary tract, and skin cancers. In lung cancer, high AAT expression correlates with advanced stage, epithelial-to-mesenchymal transition (EMT) and poor prognosis [12,13,14,15,16,17,18,19]. Similarly, increased AAT expression in intrahepatic cholangiocarcinoma is associated with more aggressive clinical features and advanced staging [20]. In breast cancer, AAT exhibits molecularly distinct effects: while native AAT suppresses proliferation and IL-6 production, its C-terminal fragment enhances NF-κB activation and promotes invasive behavior, illustrating that AAT’s biological impact depends on its molecular form [21,22]. In contrast to these tumor-promoting associations, AAT deficiency (AATD) is associated with increased risk of several malignancies, including hepatocellular carcinoma, cholangiocarcinoma, skin cancer, and leukemia, likely driven by unopposed protease activity and chronic inflammation [20,23,24,25]. In line with this notion, experimental models of colorectal cancer demonstrate that exogenous AAT reduces tumor burden and intestinal inflammation, supporting a protective role in colitis-associated tumorigenesis [26,27]. Collectively, these findings highlight that AAT may act either as a tumor-promoting or tumor-suppressive factor depending on tissue context, molecular form, and inflammatory milieu. Importantly, while elevated circulating AAT levels often reflect systemic inflammation and cancer-associated acute-phase responses, tumor-localized AAT expression has been associated with less aggressive tumor growth [8] and improved patient survival [28], suggesting that the local AAT activity within the TME may exert protective effects.

Evidence in melanoma likewise support a context-dependent role for AAT. Early clinical observations by Silver et al. [29] reported elevated serum AAT concentrations in patients with advanced melanoma compared to healthy controls, with serum AAT levels correlating with tumor burden, suggesting a tumor-promoting or inflammation-associated role of AAT. Conversely, Guttman et al. demonstrated that transgenic (TG) C57BL/6 mice expressing human AAT (hAAT) under the surfactant protein C promoter exhibited suppressed growth of multiple tumors, including melanoma, compared to wild-type (WT) mice [11]. These seemingly contradictory findings likely reflect differences in AAT source (systemic vs. local), species (human vs. mice), and experimental context. Supporting a protective role within the TME, recent transcriptomic studies have reported that higher intratumoral *SERPINA1* (gene for AAT) expression correlates with improved overall survival in melanoma patients [30,31]. However, the observation that *SERPINA1* expression is higher in melanoma tissues than in normal skin highlights a paradox that underscores the need to distinguish tumor-intrinsic expression from the TME-derived AAT.

In this study, we combined analyses of human melanoma transcriptomic datasets, in vivo tumor models, and in vitro studies using human and mouse melanoma cells to elucidate the role of AAT in melanoma. We demonstrated that *SERPINA1* expression increases with melanoma progression and that high *SERPINA1* expression in tumor tissues correlates with better overall survival in metastatic melanoma patients and confirmed the finding using another dataset. Using hAAT TG mice and in vitro studies, we reveal that AAT inhibits B16F10 mouse melanoma growth, and this effect is depending on CD8^+^ T cells rather than direct tumor cytotoxicity. Mechanistically, we demonstrate that AAT promotes melanocyte differentiation by upregulating melanocyte differentiation markers, MITF and TYR, and melanoma-associated antigens, PMEL and MART-1, which may enhance tumor immunogenicity and facilitate immune recognition. Collectively, our results identify hAAT as an indirect antitumor regulator that promotes melanocyte differentiation and augments immune-mediated tumor rejection.

## 2. Materials and Methods

### 2.1. Bioinformatics Analysis

To understand the gene expression of *SERPINA1* in human tissues, four independent datasets, GSE15605 [32], GSE7553 [33], GSE46517 [34], and GSE114445 [35], were retrieved from the Gene Expression Omnibus (GEO) database. Clinical information of 331 adult melanoma patients from The Cancer Genome Atlas (TCGA) dataset was retrieved from the associated published study [36]. Among these, 286 patients, including 42 with primary melanoma and 244 with metastatic melanoma, had complete clinicopathological information. For the survival analysis of these patients, normalized RNA-seqV2 level 3 *SERPINA1* expression data from 244 metastatic melanoma patients were retrieved from the cBioPortal database [37,38]. The Swedish dataset GSE65904 including 210 metastatic melanoma cases with complete clinical data [39] was also retrieved for patient survival analysis.

### 2.2. Mice

WT C57BL/6 mice were purchased from Jackson Laboratories (Bar Harbor, ME, USA). hAAT-TG mice, with a C57BL/6 background strain, were engineered as described [40] and provided by Dr. Charles Dinarello laboratory at the University of Colorado AMC. Circulating levels of hAAT in the TG mice were approximately 1.4 nM (calculated based on a molecular weight of 52 kDa) by a specific ELISA method, as previously described [41]. The experimental manipulations were approved by the Institutional Animal Care and Use Committee of the University of Colorado AMC under the protocol numbers B-63211(10)1E (6 October 2011) and 00282 (30 September 2020).

### 2.3. Reagent

BAY 11-7082 was obtained from Selleckchem.com (Selleckchem, Houston, TX, USA). Before use, it was reconstituted in DMSO (Corning, New York, NY, USA).

### 2.4. hAAT

Human plasma-derived AAT and human serum albumin were obtained from Athens Research & Technology (Athens, GA, USA). Before use, they were reconstituted in PBS for in vitro experiments. Human serum albumin was used at a concentration of 50 µg/mL in the group with 0 µg/mL AAT. For recombinant AAT treatment, cells were treated with 0, 10, 50, or 250 µg/mL, concentrations selected to reflect physiologically relevant levels.

### 2.5. Cell Lines

Human melanoma cell lines (A375, 1205Lu, SK-MEL-28, HS294T, and 451Lu) and mouse melanoma cell line B16F10 were from the American Type Culture Collection (Manassas, VA, USA). Cells were grown in RPMI medium 1640 (Gibco, Grand Island, NY, USA) supplemented with 10% fetal bovine serum (Gemini Bio-Products, West Sacramento, CA, USA) and tested for mycoplasma contamination monthly.

### 2.6. Tumor Formation In Vivo and Treatment

0.1 × 10^6^ B16F10 cells were suspended in Matrigel Matrix (Corning, Bedford, MA, USA) diluted 1:1 with ice-cold RPMI-1640 and injected subcutaneously into both flanks of WT mice.

In an additional experiment, to assess the effects of hAAT on specific T-cell subsets (CD4^+^ vs. CD8^+^ T cells) within the TME, hAAT-TG mice were depleted of CD4^+^, CD8^+^, or both CD4^+^/CD8^+^ T cells by intraperitoneally injecting 250 μg of Ultra-LEAF purified anti-mouse CD4 (GK1.5), CD8α (53–6.7), or rat IgG2b isotype control (RTK4530) antibodies (BioLegend, San Diego, CA, USA) on days −3, 0, and 3 relative to tumor implantation (day 0). Subsequent weekly doses were administered on days 7, 14, and 21 after tumor implantation.

Tumor growth was monitored per 2–4 days with an electronic digital caliper, and tumor volume was calculated according to the formula: tumor volume (mm^3^) = longest diameter × shortest diameter^2^/2. At the end of the experiments, tumor tissues were harvested. Mice were also monitored for body condition and signs of discomfort. Humane endpoints were applied in accordance with IACUC guidelines established the University of Colorado. The experiments were terminated when tumors reached 1000 mm^3^ volume.

### 2.7. Immunohistochemistry and Terminal Deoxynucleotidyl Transferase-dUTP Nick End Labeling (TUNEL) Assay

Tumor tissues were fixed in 10% neutral buffered formalin. After paraffin embedding, tumor specimens were cut into 5-μm sections and stained with hematoxylin and eosin. Immunohistochemistry was performed as previously described [42]. Sections were stained with specific antibodies, followed by AEC HRP substrate (Vector Laboratories, Newark, CA, USA). Primary antibodies included rabbit anti-mouse Ki-67 (Abcam, Cambridge, MA, USA), rat anti-mouse CD3 (Bio-Rad, Hercules, CA, USA), rat anti-mouse CD8a antibody (ThermoFisher, Waltham, MA, USA), rabbit anti-mouse CD4 (Abcam), and rat anti-mouse Foxp3 (ThermoFisher). TUNEL assay kit was from Abcam and performed following its standard protocols. Quantitative analyses of mitotic figures, Ki-67^+^ cells, and CD3^+^ cells were performed by cell counting at 40× magnification using the Aperio ImageScope viewing software (version 12.4, Leica Biosystems, Deer Park, IL, USA). Quantitative analyses of CD4^+^, CD8^+^, and Foxp3^+^ positive cells were performed by cell counting at 40× magnification using the EVOS Cell Imaging Systems (ThermoFisher).

### 2.8. Cell Viability Assay

Cells were seeded into a 96-well plate at a density of 1 × 10^3^ cells per well and allowed to adhere for 2 h before being exposed to daily dose of each concentrations of hAAT or H_2_O_2_ for 72 h. Cell viability was measured using the CellTiter 96 AQueous One Solution Cell Proliferation Assay kit (Promega, Madison, WI, USA).

### 2.9. Semi-Solid Sphere Formation Assay

Cells were trypsinized, washed with PBS, and resuspended at a density of 1000 cells/mL in a 1:1 mixture of DMEM sphere medium and methylcellulose medium. Sphere medium consisted of DMEM/F12 (Hyclone, Logan, UT, USA) supplemented with B27 (Invitrogen, Carlsbad, CA, USA), 20 ng/mL EGF, 20 ng/mL bFGF (BD Biosciences, Franklin Lakes, NJ, USA), and 4 µg/mL heparin (Sigma, St. Louis, MO, USA). AAT was added at the indicated concentrations, and the cell suspension was plated onto polyHEMA-coated 6-well plates. After 10 days, the total number of spheres per well was counted. All assays were performed in triplicate wells.

### 2.10. Western Blot

Cells were washed in phosphate-buffered saline and lysed in RIPA buffer (Sigma) containing 1% (*v*/*v*) Halt protease and phosphatase inhibitor cocktail (Thermo Scientific, Rockford, IL, USA). Cell lysates were separated on 4–15% Mini-PROTEAN TGX precast gels (Bio-Rad), followed by electrotransfer onto PVDF membranes. After blocking in 5% nonfat milk, the immunoblots were incubated with primary antibodies and then with a horseradish peroxidase-conjugated secondary antibody (Sigma). The primary antibodies included mouse anti-TYR (Santa Cruz Biotechnology, Dallas, TX, USA), rabbit antibodies against MITF (Abcam), melanoma gp100 (Abcam), MART-1 (Novus Bio, Centennial, CO, USA), NFκB p65 (Cell Signaling Technology, Danvers, MA, USA), phosphor NFκB p65 (Cell Signaling Technology) and cyclophilin A (CyPA) (Cell Signaling Technology). Signals were visualized by SuperSignal West Femto Maximum Sensitivity Substrate (Thermo Scientific) and analyzed using the Odyssey imaging system (LI-COR, Lincoln, NE, USA). Original western blots can be found in Supplementary Materials.

### 2.11. Quantitative RT-PCR

Total RNA was isolated using TRIzol reagent (Invitrogen) and reverse transcribed using MMLV reverse transcriptase (Promega). qPCR was performed with the PowerUp SYBR Green PCR Master Mix (Applied Biosystems, Foster City, CA, USA) on the AriaMx Real-time PCR System (Agilent, Santa Clara, CA, USA). For the mouse cell studies, we used the following primers: *Mitf*, 5′-GCAAGAGGGAGTCATGCAGT-3′ (forward) and 5′-AGAACTGCTGCTCTTCAGAGGT-3′ (reverse); *Tyr*, 5′-CTAACTTACTCAGCCCAGCATC-3′ (forward) and 5′-GGGTTTTGGCTTTGTCATGG-3′ (reverse); *Pmel*, 5′-CACAAGCAACAACCACAGAG-3′ (forward) and 5′-CGATATAGAACACAGTCCAGGG-3′ (reverse); *Mlana*, 5′-AAGAGAAATCCCATCAGCCC-3′ (forward) and 5′-TTAAAGCGGAAGTGTGAGGG-3′ (reverse); and *Gapdh*; 5′-CTCATGACCACAGTCCATGC-3′ (forward) and 5′-CACATTGGGGGTAGGAACAC-3′ (reverse). For the human cell studies, we used the following primers: *MITF*, 5′-CGGGAACAGGACCATGGTTA-3′ (forward) and 5′-AGCTAGCCCCTGAAATGAATCC-3′ (reverse); *TYR*, 5′-TCATCCAAAGATCTGGGCTATG-3′ (forward) and 5′-CCAAGGAGCCATGACCAG-3′ (reverse); *PMEL*, 5′-TTTGGTTGCTGGAGGGAAG-3′ (forward) and 5′-TGGTTCTGAGTTGCCTTGAG-3′ (reverse); *MLANA*, 5′-ATGCGAAGAGAAGATGCTCA-3′ (forward) and 5′-AGCATGTCTCAGGTGTCTCG-3′ (reverse); and *GAPDH*, 5′-TGCACCACCAACTGCTTAGC-3′ (forward) and 5′-GGCATGGACTGTGGTCATGAG-3′ (reverse).

### 2.12. Melanin Measurement

Each melanoma cell line was seeded in 6-well plates at a density of 2 × 10^5^ cells/well and exposed to a dose of each hAAT concentration for 24 h. Cells were trypsinized, collected, and centrifuged at 2000× *g* for 5 min. The cell pellets were dissolved in 1 N sodium hydroxide for 30 min at 80 °C to extract intracellular melanin, which was measured by absorbance at 405 nm. Melanin levels were calculated by referring to a calibration curve obtained with synthetic melanin (Sigma). To normalize intracellular melanin, we measured the total protein amount with Pierce Rapid Gold BCA Protein Assay Kit (Thermo Scientific) in the same cell samples and divided the melanin concentration by the total protein amount.

### 2.13. Statistical Analysis

GraphPad Prism 9 (GraphPad Software, La Jolla, CA, USA) was used for statistical analysis. Kaplan–Meier method was used to create survival curves, and the log-rank test was used for statistical differences between two patient groups. Experimental numerical data are expressed as mean ± standard error (SEM). The two-tailed Student’s *t*-test was used for comparison between two groups, while for comparison among more groups, one-way ANOVA with Dunnett’s multiple comparison test was used. A value *p* < 0.05 was considered statistically significant.

## 3. Results

### 3.1. SERPINA1 Expression Increases with Melanoma Progression and Associates with Improved Overall Survival in Metastatic Melanoma Patients

**Figure 1:**
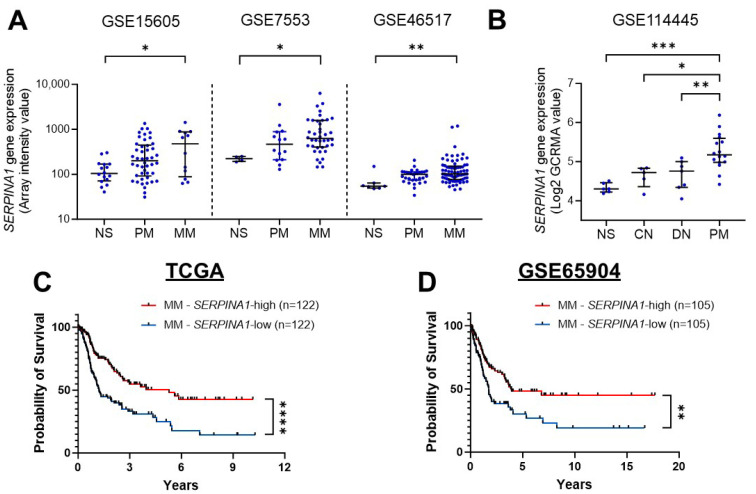
*SERPINA1* expression and Kaplan–Meier survival analysis of melanoma patients. (**A**,**B**) Expression levels of *SERPINA1* in normal skin, common nevus, dysplastic nevus, primary melanoma, and metastatic melanoma, analyzed using GEO datasets (**A**) GSE15605, GSE7553, GSE46517, and (**B**) GSE114445. Data are presented as median ± interquartile range; statistical significance was determined by the Mann–Whitney test. (**C**) Kaplan–Meier overall-survival curves for patients with metastatic melanoma in the TCGA-SKCM cohort. (**D**) Validation of the survival association in the GSE65904 metastatic melanoma cohort. Patients with complete clinicopathological data were dichotomized into *SERPINA1*-high and -low groups based on their normalized RNA-seq expression levels. NS, normal skin; CN, common nevus; DN, dysplastic nevus; PM, primary melanoma; MM, metastatic melanoma * *p* < 0.05, ** *p* < 0.01, *** *p* < 0.001, and **** *p* < 0.0001.

If AAT is protective, its expression would be expected to decrease with disease advancement. To clarify the clinical relevance of AAT in melanoma, we first examined *SERPINA1* expression across publicly available transcriptomic datasets. Consistent with previous reports showing elevated *SERPINA1* levels in melanoma compared with normal skin [30,31], our integrated analysis of multiple GEO datasets (GSE15605, GSE7553, GSE46517, and GSE114445) demonstrated a progressive increase in *SERPINA1* expression from normal skin to common nevus, dysplastic nevus, primary melanoma, and finally metastatic melanoma (Figure 1A,B).

This paradoxical increase prompted us to assess whether the expression pattern has prognostic significance in primary and metastatic melanoma subsets from TCGA dataset. Among primary melanoma cases (*n* = 42), Kaplan–Meier analysis showed no significant difference in overall survival between patients with high and low *SERPINA1* expression (Figure S1). In contrast, within the metastatic melanoma cohort, patients with higher *SERPINA1* expression exhibited significantly improved overall survival compared with those with lower expression (*p* < 0.0001) (Figure 1C). This association was further validated in an independent metastatic melanoma cohort (GSE65904), which demonstrated a similar survival advantage for *SERPINA1*-high tumors (Figure 1D).

Together, these findings confirm that *SERPINA1* expression increases as melanoma progresses but that its prognostic benefit emerges specifically in metastatic disease. These findings suggest that AAT plays a context-dependent, potentially immunomodulatory role within the metastatic melanoma microenvironment, supporting its investigation as a tumor-suppressive and immune-regulatory factor.

### 3.2. Exogenous AAT Does Not Directly Affect Melanoma Cell Viability

The paradoxical pattern observed in Figure 1, in which *SERPINA1* expression increases with melanoma progression yet correlates with improved prognosis, suggests that AAT expression within the TME may originate from non-tumor sources rather than melanoma cells themselves. Supporting this interpretation, prior transcriptomic analyses have reported that *SERPINA1* expression in melanoma tissues is predominantly contributed by tumor-infiltrating immune cells, particularly macrophages and lymphocytes, rather than by melanoma cells [30,31]. Public transcriptomic and single-cell RNA-sequencing data in the Human Protein Atlas further confirm that *SERPINA1* expression is enriched in infiltrating immune cell populations rather than in tumor cells themselves (available at: https://www.proteinatlas.org, accessed on 10 October 2025). Consistent with these findings, *SERPINA1* transcript levels are undetectable or very low in normal skin and in melanoma cell lines (including A375 and SK-MEL-28), whereas the expression is markedly higher in liver and lung cancer cell lines. Thus, elevated intratumoral *SERPINA1* expression most likely reflects increased infiltration of immune cells or stromal cells rather than tumor-intrinsic AAT production.

**Figure 2:**
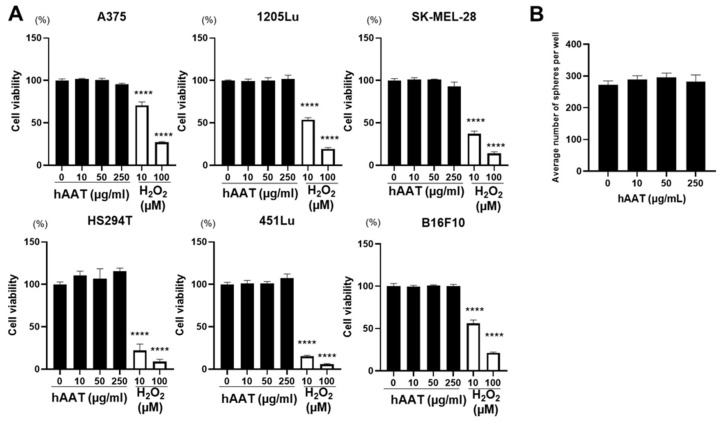
Cell viability of human metastatic melanoma cell lines treated with human AAT (hAAT). (**A**) Human metastatic melanoma cell lines (A375, 1205Lu, SK-MEL-28, HS294T, and 451Lu) and mouse metastatic melanoma cell line (B16F10) were treated with hAAT at the indicated concentrations (0–250 µg/mL). H_2_O_2_ was used for a positive control. Cell viability was measured using an MTS assay after 72 h of hAAT treatment. (**B**) Bar diagram showing the colony number per well for each group. Data are expressed as the mean ± SEM (*n* = 3). **** *p* < 0.0001 vs. control (without treatment).

Given that higher AAT levels within the melanoma TME are associated with better patient prognosis, we hypothesized that AAT secreted by non-tumor cells might exert tumor-inhibitory effects on melanoma growth. In humans, circulating AAT concentrations range from 1.0–2.0 mg/mL under basal conditions and can increase up to 4-fold during acute-phase responses [43,44,45]. Tissue and interstitial AAT concentrations, including those reported in inflamed skin and solid tumors, are typically estimated to be 10% of plasma levels (approximately 100–200 µg/mL) and may exceed 1 mg/mL in highly inflamed TME. Accordingly, the 10–250 µg/mL concentration employed in our in vitro experiments fall well within the endogenous range encountered in human skin and tumor tissues and remains sub-physiological relative to circulating plasma levels. Because melanoma cells express low to no *SERPINA1* (available at https://www.proteinatlas.org), melanoma cells are physiologically exposed to AAT primarily as an extracellular protein secreted by immune and stromal cells. Therefore, we tested whether exogenous AAT could directly influence melanoma cell proliferation or survival in vitro. Treatment of multiple human melanoma cell lines and B16F10 mouse melanoma cells with hAAT across a range of concentrations (0–250 μg/mL) for 72 h did not alter cell viability compared to untreated controls (Figure 2A), indicating that hAAT had no direct cytotoxic or cytostatic effects on melanoma cells in vitro. To examine whether AAT influences other properties relevant to melanoma progression, we next assessed anchorage-independent growth using a semi-solid sphere formation assay on B16F10 cells. Exogenous AAT did not significantly alter the number of colonies, spheroid size, or colony formation efficiency into the surrounding matrix (Figure 2B and Figure S2), suggesting that AAT does not directly modulate anchorage-independent clonogenic growth in this model. Together, these findings support the conclusion that the favorable prognostic association of AAT in melanoma is unlikely to be explained by direct tumor cell effects and instead reflects its indirect role within the TME.

### 3.3. hAAT Strongly Inhibits Melanoma Tumor Growth in hAAT-TG Mice

Because exogenous administration of hAAT did not produce a significant tumor-suppressive effect in vitro, we next investigated whether hAAT could modulate melanoma growth in vivo in the TME. However, direct evaluation of human melanoma cells in vivo would require immunodeficient nude mice, in which immune-mediated antitumor responses cannot be assessed. Therefore, we used hAAT-TG mice, in which hAAT is expressed in surfactant protein C-positive lung cells and secreted into the circulation, to investigate the impact of hAAT on melanoma development.

**Figure 3:**
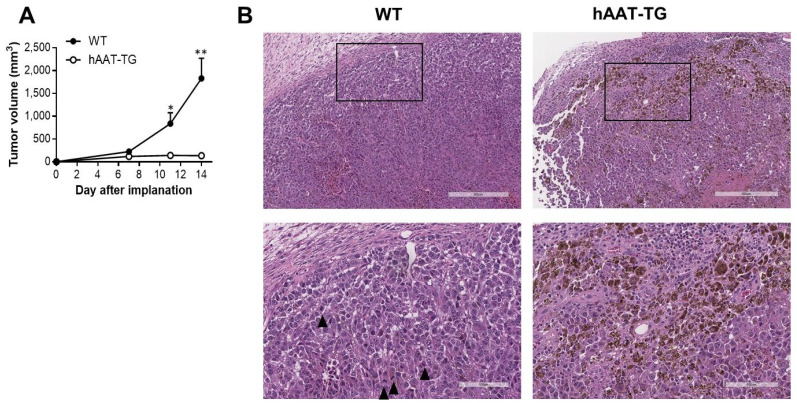
Effect of hAAT on mouse melanoma. (**A**) Tumor growth of B16F10 (0.1 × 10^6^) in WT or hAAT-TG mice monitored over 14 days. (**B**) Hematoxylin and eosin (H&E) staining of B16F10 tumors derived from WT and hAAT-TG mice shown in (**A**). Trianglesindicate mitotic figures. (Upper panels) low-magnification images. (Lower panels) high-magnification images (black frames). Scale bar = 300 μm (upper panel) or 100 μm (lower panel). Far right panel: Quantification of mitotic figures. Data represent mean ± SEM (*n* = 6 (**A**), 8 (**B**)). * *p* < 0.05 and ** *p* < 0.01 vs. corresponding tumors in WT mice on the same day.

To evaluate the impact of hAAT on melanoma growth in vivo, B16F10 melanoma cells were injected subcutaneously into WT and hAAT-TG mice, and tumor volumes were monitored for 14 days. Tumor growth was significantly suppressed in hAAT-TG mice compared with WT controls, with mean tumor volumes remaining below 140 mm^3^, whereas tumor in WT mice exceeded 1800 mm^3^ on day 14 (*p* < 0.01) (Figure 3A). Histopathologic examination of B16F10 tumors demonstrated smaller tumors with reduced mitotic activity, enhanced pigmentation, and increased lymphocytic infiltration at the tumor periphery in hAAT-TG mice compared with WT mice (Figure 3B).

### 3.4. hAAT Reduces Tumor-Cell Proliferation and Enhances Apoptosis and T-Cell Infiltration in hAAT-TG Mice

**Figure 4:**
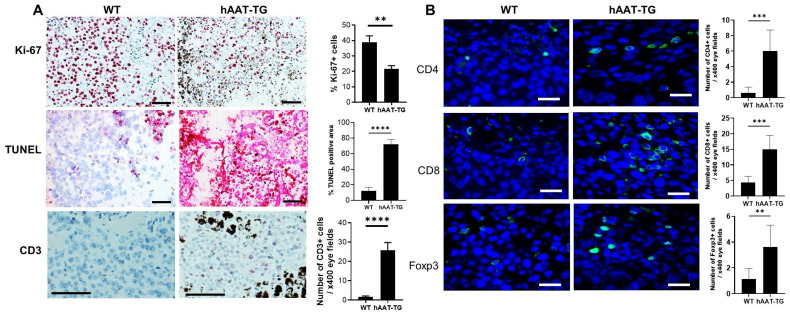
Immunohistochemical and immunofluorescent staining of B16F10 tumors from WT and hAAT-TG mice. (**A**) Representative tumor sections from Figure 3B were stained for Ki-67, apoptosis using TUNEL assay, and CD3 with 3-Amino-9-ethylcarbazole (AEC) staining. Left panels: sections from WT mice. Middle panels: sections from hAAT-TG mice. Right panels: quantification of Ki-67-positive cells, TUNEL-positive area, and number of CD3^+^ cells. (**B**) Representative tumor sections from Figure 3B were stained for CD4, CD8, and Foxp3 with Immunofluorescence staining (Green). Left panels: sections from WT mice. Middle panels: sections from hAAT-TG mice. Right panels: quantification of CD4^+^ cells, CD8^+^ cells, and Foxp3^+^ cells. Representative images are shown. Scale bar = 100 μm. ** *p* < 0.01, *** *p* < 0.001, and **** *p* < 0.0001 vs. corresponding tumors in WT mice on the same day.

To explore mechanisms underlying the marked reduction in melanoma tumor growth observed in hAAT-TG mice, we next examined tumor-cell proliferation, apoptosis, and lymphocytic infiltration. Tumor sections in Figure 3B were stained for Ki-67 (a proliferation marker), apoptotic cells using the TUNEL assay, and CD3 (a pan-T cell marker) (Figure 4A).

Quantitative analysis showed that the proportion of Ki-67-positive tumor cells was reduced to approximately 55% of that in WT tumors, consistent with the 56% reduction in mitotic figures noted earlier. In contrast, TUNEL staining was markedly increased in hAAT-TG tumors, revealing widespread apoptotic cells characterized by larger nuclei and diffuse intratumoral distribution, whereas WT tumors exhibited only scattered TUNEL-positive cells. Moreover, CD3-positive lymphocytes were increased by ~15.6-fold in hAAT-TG tumors compared with WT controls, indicating infiltration of T cells into the TME. To further investigate T-cell infiltration, we performed immunofluorescence staining for CD3, CD4, CD8, and Foxp3 in tumor sections. Quantitative analysis revealed that hAAT-TG mice exhibited significantly increased numbers of CD4^+^, CD8^+^, and Foxp3^+^ T cells within the TME compared with WT mice (Figure 4B), indicating enhanced recruitment of multiple T-cell subsets associated with AAT expression.

The distinct morphology and distribution of TUNEL^+^ and CD3^+^ cells suggest that the majority of apoptotic signal arises from tumor cells rather than infiltrating lymphocytes. Collectively, these data demonstrate that hAAT expression is associated with decreased tumor-cell proliferation, enhanced apoptosis, and pronounced T-cell infiltration. While reduced proliferation may contribute, the strong association between hAAT expression, increased T-cell infiltration, and enhanced apoptosis is consistent with a potential role for immune-mediated cytotoxicity in hAAT-associated tumor suppression.

### 3.5. CD8^+^ T-Cell Depletion Enhances Tumor Growth, Whereas CD4^+^ T-Cell Depletion Reduces Tumor Growth in hAAT-TG Mice

**Figure 5:**
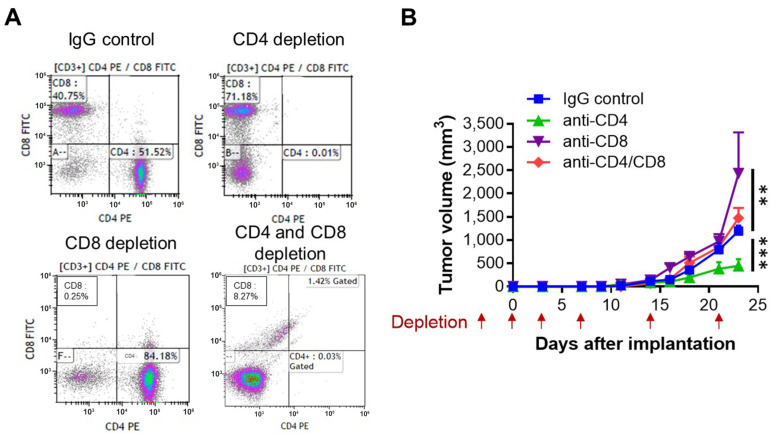
Tumor growth curves of hAAT-TG mice depleted of CD4^+^ and/or CD8^+^ T cells. (**A**) hAAT-TG mice were depleted of CD4^+^, CD8^+^, or both CD4^+^/CD8^+^ T cells using specific depleting antibodies. Flow cytometric analysis of CD4 and CD8 after CD3-gating of splenocytes 3 days after the depletion in mice treated with IgG control, anti-CD4, anti-CD8, and combination of anti-CD4 and anti-CD8 antibodies. (**B**) hAAT-TG mice were depleted of CD4^+^, CD8^+^, or both CD4^+^/CD8^+^ T cells using specific depleting antibodies on days −3, 0, and 3 relative to tumor implantation (day 0), followed by weekly doses on days 7, 14, and 21 (red arrows). Tumor growth was monitored for 24 days. Data are represented as mean ± SEM (*n* = 4 for mice with IgG control, anti-CD4, and anti-CD8, and *n* = 2 for mice treated with anti-CD4 and anti-CD8). ** *p* < 0.01, *** *p* < 0.001.

Given the increased CD3^+^ T-cell infiltration and enhanced apoptosis observed in hAAT-TG tumors, we next tested whether hAAT-mediated tumor suppression depends on specific T-cell subsets. To this end, hAAT-TG mice were treated with depleting antibodies against CD4^+^, CD8^+^, or both CD4^+^/CD8^+^ T cells during B16F10 tumor implantation (Figure 5).

Compared to TG mice treated with IgG control, CD8^+^ T-cell depletion significantly accelerated B16F10 tumor growth, demonstrating CD8^+^ cytotoxic T cells are essential for the antitumor effect of hAAT. In contrast, CD4^+^ T-cell depletion led to a significant reduction in tumor growth, suggesting that CD4^+^ T cells support, rather than oppose, melanoma progression under these conditions. Notably, dual depletion of CD4^+^ and CD8^+^ T cells resulted in tumor growth kinetics intermediate between those observed with single CD8^+^ T cell depletion and the single CD4^+^ T cell depletion. Although this pattern suggests a potential modulatory role of CD4^+^ T cells in shaping CD8^+^ T-cell-mediated antitumor responses, the limited sample size (*n* = 2) precludes definitive conclusions, and these data with double depletion are therefore presented as descriptive.

Together, these findings indicate that hAAT promotes antitumor immunity primarily through CD8^+^ T-cell–mediated cytotoxicity, while CD4^+^ T cells appear to play a distinct, context-dependent role in regulating tumor growth.

### 3.6. hAAT Enhances Melanocytic Differentiation and Pigmentation in Melanoma Cells

While previous results establish that hAAT suppresses melanoma growth through immune-dependent mechanisms, particularly involving CD8^+^ cytotoxic T cells, the magnitude of tumor inhibition observed in hAAT-TG mice was strikingly greater than what could be explained by immune activation alone, suggesting that additional hAAT-driven changes within melanoma cells may further enhance antitumor immunity. Because AAT can influence cellular differentiation in other tissues, we hypothesized that hAAT affects melanoma differentiation and pigment production, processes that can increase melanoma-associated antigen expression and promote immune recognition.

**Figure 6:**
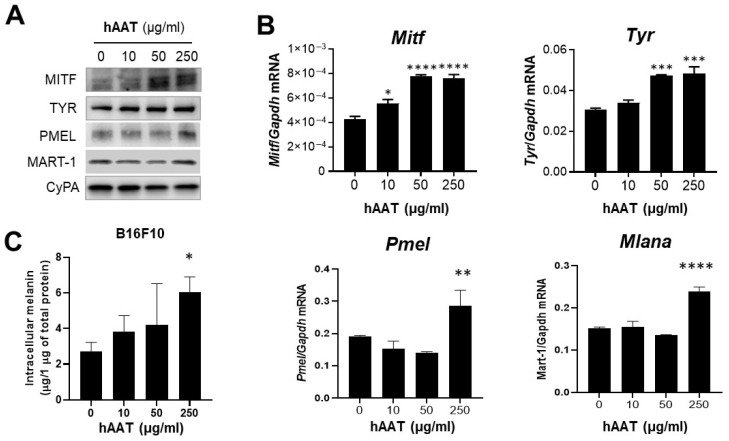
Effect of hAAT on pigmentation regulators in B16F10 cells. B16F10 cells were treated with indicated concentrations of hAAT for 24 h. (**A**) Western blot of MITF, TYR, PMEL, and MART-1 protein. (**B**) qRT-PCR of *Mitf*, *Tyr*, *Pmel*, and *Mlana* mRNA levels, normalized to *Gapdh*. (**C**) Intracellular melanin content in B16F10 cells after hAAT treatment. Representative images are shown or data are expressed as mean ± SEM (*n* = 3). * *p* < 0.05, ** *p* < 0.01, *** *p* < 0.001, and **** *p* < 0.0001 vs. untreated control.

Histologic examination of B16F10 tumors from hAAT-TG mice revealed visibly enhanced melanin pigmentation compared with tumors from WT mice (Figure 3B and Figure 4), suggesting that hAAT might promote melanocytic differentiation in vivo. To determine whether this reflects a direct effect of hAAT on melanoma cells, we treated B16F10 cells with 10–250 µg/mL hAAT for 24 h and evaluated the gene and protein expressions of MITF, TYR, PMEL, and MART-1—key regulators of melanin biosynthesis (Figure 6). hAAT exposure increased the protein expression of all three factors by at least 1.6-fold (Figure 6A) and significantly upregulated the gene expression of *Mitf*, *Tyr*, *Pmel*, and *Mlana* (Figure 6B). Since these proteins are key regulators of melanin synthesis, we next measured intracellular melanin content in B16F10 cells treated with 10–250 µg/mL hAAT for 72 h (Figure 6C). Exposure to 250 µg/mL hAAT increased melanin levels by approximately 2.2-fold compared with untreated controls, confirming that hAAT directly enhances pigment production in melanoma cells.

**Figure 7:**
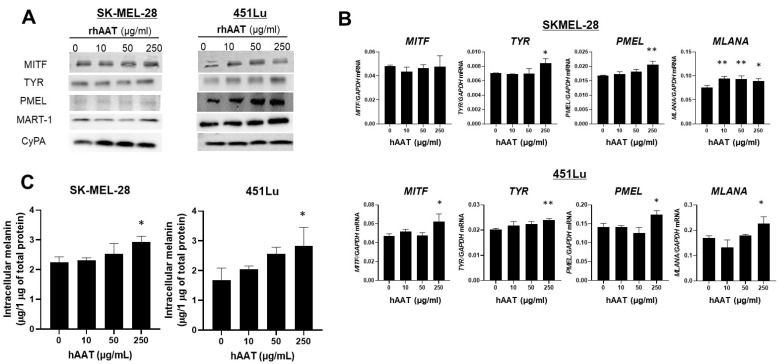
Effect of hAAT on pigmentation regulators in human melanoma cells. Human melanoma cell lines, SK-MEL-28 and 451Lu, were treated with indicated concentrations of hAAT for 24 h. (**A**) Western blot of MITF, TYR, PMEL, and MART-1 protein. (**B**) qRT-PCR of *MITF*, *TYR*, *PMEL*, and *MLANA* mRNA levels, normalized to *GAPDH*. (**C**) Intracellular melanin content in SK-MEL-28 and 451Lu cells after hAAT treatment. Data are presented as mean ± SEM (*n* = 3). * *p* < 0.05 and, ** *p* < 0.01 vs. untreated control.

To determine whether this differentiation-promoting effect extends to human melanoma, we next treated SK-MEL-28 and 451Lu cell lines, both harboring the *BRAF^V600E^* mutation and producing intracellular melanin, with 10, 50, or 250 µg/mL hAAT for 72 h. hAAT treatment increased the protein expression of MITF, TYR, PMEL, and MART-1 (Figure 7A) and significantly upregulated their corresponding mRNA levels (Figure 7B). Consistent with these molecular changes, intracellular melanin content increased by at least 1.4-fold in SK-MEL-28 and 1.7-fold in 451Lu cells exposed to 250 µg/mL hAAT (Figure 7C).

Together, these findings demonstrate that hAAT promotes melanocytic differentiation pigment synthesis in both murine and human melanoma cells. The resulting upregulation of pigmentation-associated antigens suggests that hAAT may enhance tumor immunogenicity by increasing melanoma antigen expression and thereby potentiating T-cell-mediated cytotoxicity.

## 4. Discussion

Our study reveals that hAAT exerts an antitumor effect in melanoma primarily through immune-mediated and differentiation-dependent mechanisms, rather than direct cytotoxicity. Transcriptomic analyses demonstrated that *SERPINA1* expression rises with melanoma progression but paradoxically correlates with improved overall survival in metastatic disease. These observations suggest that AAT functions as a context-dependent regulator within the TME. In hAAT-TG mice, we show that physiologic AAT expression markedly suppressed melanoma growth, which was dependent on CD8^+^ T-cell activity and accompanied by enhanced melanocytic differentiation. Collectively, these data identify AAT as an immunomodulatory and differentiation-inducing molecule that reprograms the melanoma TME toward immune activation.

Consistent with our observations, previous transcriptomic studies also reported that higher *SERPINA1* expression is associated with favorable outcomes in cutaneous melanoma, consistent with our findings [30,31]. Analysis of the TCGA and TIMER databases have shown enrichment of B cells, CD4^+^ T cells, and CD8^+^ T cells in *SERPINA1*-high melanomas [30,31], suggesting that hAAT expression correlates with an inflamed or immune-active TME. In contrast, *SERPINA1* mutations, primarily missense variants, are linked to poor prognosis [18], highlighting the functional importance of intact AAT protein. Moreover, Guttman et al. reported that AAT promotes CD8^+^ T-cell migration into melanoma [9], supporting the concept that hAAT facilitates immune infiltration rather than immune escape. Together, these studies and our in vivo findings support the view that AAT acts as an immune adjuvant enhancing cytotoxic T-cell-mediated antitumor immunity.

The immune dependence of hAAT-mediated tumor inhibition was confirmed by selective T-cell depletion. Loss of CD8^+^ T cells abolished tumor control, whereas depletion of CD4^+^ T cells enhanced suppression, suggesting that AAT augments cytotoxic activity while limiting Treg-mediated suppression. These findings align with previous work showing that AAT modulates macrophage polarization toward an M1-like phenotype and supports effector T-cell survival [11]. Collectively, these results indicate that AAT promotes a more immunostimulatory microenvironment that favors tumor elimination. Such immune-modulating properties may partly explain the improved prognosis observed in patients with *SERPINA1*-high melanomas.

Another key finding of this study is that hAAT promotes melanocytic differentiation and pigment synthesis, reflected by the upregulation of MITF, TYR, PMEL (gp100), and MART-1. MITF governs melanocyte lineage commitment and coordinates pigment-gene expression [19], and its activation often signifies a transition from proliferative to differentiated states. Because gp100 and MART-1 contain immunodominant CD8^+^ T-cell epitopes [46,47], AAT-induced melanocytic differentiation likely enhances tumor immunogenicity. This suggests that AAT may not only suppress tumor growth indirectly but may also increase the antigenic visibility of melanoma cells to immune surveillance, thereby linking differentiation with immune activation. Consistent with this interpretation, AAT is well known for its anti-inflammatory properties, including attenuation of NF-κB signaling [48,49], a pathway that has been implicated in suppressing melanocytic differentiation. In support of this connection, pharmacological inhibition of NF-κB resulted in increased MITF expression, and a similar upregulation of MITF was observed in melanoma cells treated with AAT (Figure S3). These observations suggest that AAT may promote MITF expression, at least in part, through modulation of NF-κB-associated inflammatory signaling. By influencing MITF and its downstream melanogenesis-related genes, AAT may contribute to a shift in melanoma cells toward a more differentiated and immunogenic phenotype, providing a plausible mechanistic link between NF-κB inhibition, melanocytic differentiation, and tumor growth suppression. However, we emphasize that this pathway remains associative, and further genetic or pathway-specific studies will be required to establish definitive causality.

Clinically, the role of AAT in cancer is complex and context-dependent. Elevated serum AAT levels are commonly associated with poor prognosis in hepatocellular [16], pancreatic [30], and lung cancers [31], likely reflecting systemic inflammation. In contrast, tumor-localized AAT expression is often favorable, as seen in melanoma, where higher *SERPINA1* expression correlates with improved survival. These opposing associations may depend on tissue source and local concentration: hepatic AAT production contributes to systemic inflammation, whereas immune-cell-derived AAT within the TME may restrain protease-driven tissue damage and support antitumor immunity. Tumor heterogeneity further complicates interpretation, as *SERPINA1* can be expressed by macrophages, stromal cells, and occasionally tumor cells [30]. The balance between these cellular sources likely determines whether AAT acts as a protective or permissive factor in cancer progression.

In conclusion, our results uncover a previously unrecognized role of hAAT as a dual immunomodulatory and differentiation-promoting factor in melanoma. By enhancing CD8^+^ T-cell cytotoxicity and stimulating melanocytic differentiation, hAAT suppresses tumor growth and promotes immune-mediated rejection. These insights provide a mechanistic framework linking AAT biology to melanoma immunogenicity and suggest that therapeutic modulation of AAT or its downstream pathways could improve responses to immune-based therapies.

## 5. Conclusions

The role of AAT in cancer biology is multifaceted. Our findings identify a previously unrecognized tumor-suppressive function of hAAT in melanoma, mediated not by direct cytotoxicity but through reprogramming of the tumor microenvironment. hAAT enhances melanocytic differentiation, upregulates melanoma-associated antigens, and promotes CD8^+^ T-cell-mediated immune responses, collectively leading to marked inhibition of tumor growth. These results highlight the dual immunomodulatory and differentiation-inducing roles of hAAT and suggest that physiological or therapeutically augmented AAT activity could improve melanoma immunogenicity and antitumor immunity. Future studies are warranted to determine the therapeutic potential and optimal delivery strategies for hAAT or its downstream effectors in melanoma treatment.

## Data Availability

The data presented in this study are available in this article or associated Supplementary Material.

## Supplementary Materials

The following supporting information can be downloaded at: https://www.mdpi.com/article/10.3390/biom16010122/s1, Figure S1: Prognostic analysis of SERPINA1 expression in primary melanoma. Figure S2: Microscopic images of Figure 2B. Figure S3: MITF expression is increased in AAT-treated human and mouse melanoma cell lines via NFκB suppression. Figure S4: Original blots of Figure 6A. Figure S5: Original blots of Figure 7A.
