# Supplementary material for: Human Alpha-1 Antitrypsin Suppresses Melanoma Growth by Promoting Tumor Differentiation and CD8+ T-Cell-Mediated Immunity
Source: Biomolecules. 2026 Jan 12;16(1):122. doi: 10.3390/biom16010122 (PMC12838898; doi:10.3390/biom16010122)

**Figure S1.**

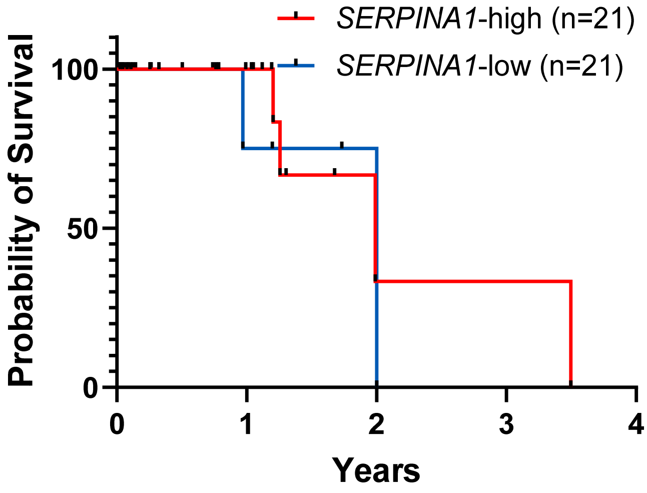


**Supplemental Figure S1.** Prognostic analysis of *SERPINA1* expression in primary melanoma. Kaplan–Meier overall-survival curve for primary melanoma cases (n = 42) in the TCGA-SKCM dataset, dichotomized into *SERPINA1*-high and *SERPINA1*-low expression groups. No statistically significant difference in overall survival was observed ($p > 0.05$, log-rank test).

**Figure S2.**

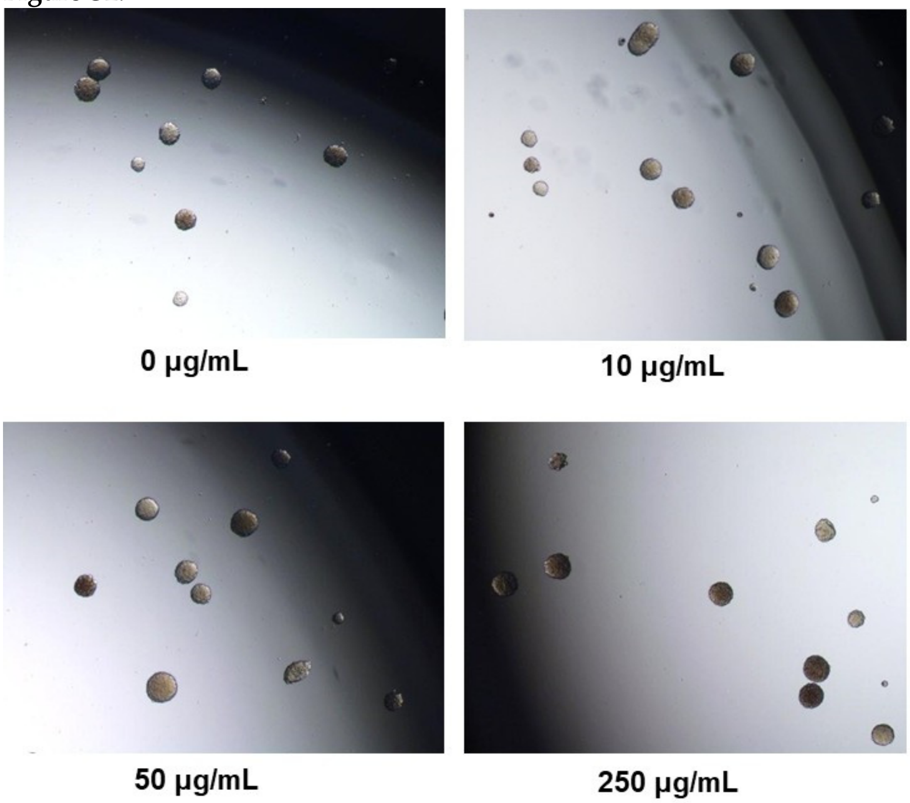


**Supplemental Figure S2.** Assessment of AAT-treated B16F10 3D colony formation efficiency. Representative images of the 3D colony formation (Semi-solid sphere formation assay) potential of each concentration of hAAT-treated B16F10.

**Figure S3**

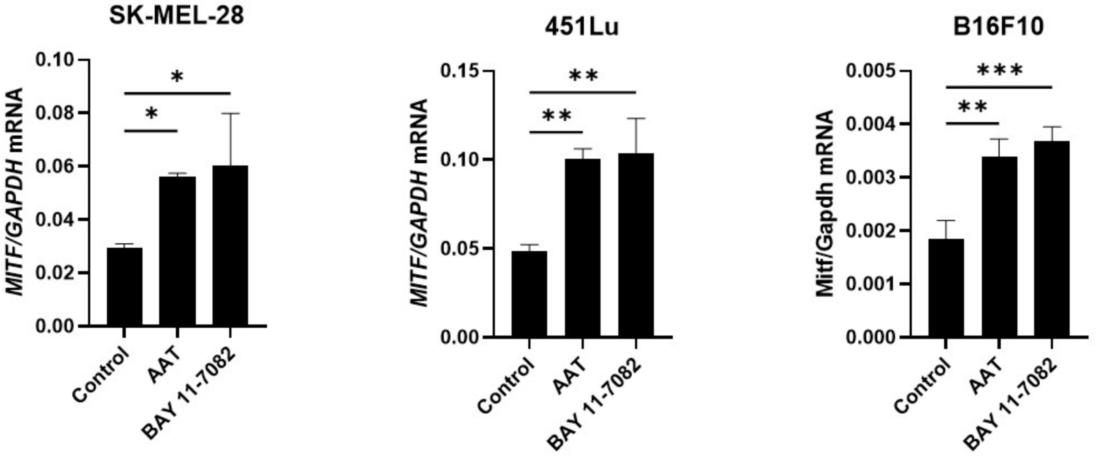


**Supplemental Figure S3. MITF expression is increased in AAT-treated human and mouse melanoma cell lines via NFκB suppression.** qRT-PCR of *MITF* (SK-MEL-28 and 451Lu cells) and *Mitf* (B16F10 cells) mRNA levels treated with 250 μg/mL AAT or NFkB inhibitor (2 μM Bay 11-7082), normalized to *GAPDH* (SK-MEL-28 and 451Lu cells) or *Gapdh* (B16F10 cells). *p < 0.05, **p < 0.01, and ***p < 0.001 vs. untreated control.

**Figure S4.** Original images of Figure 6A.

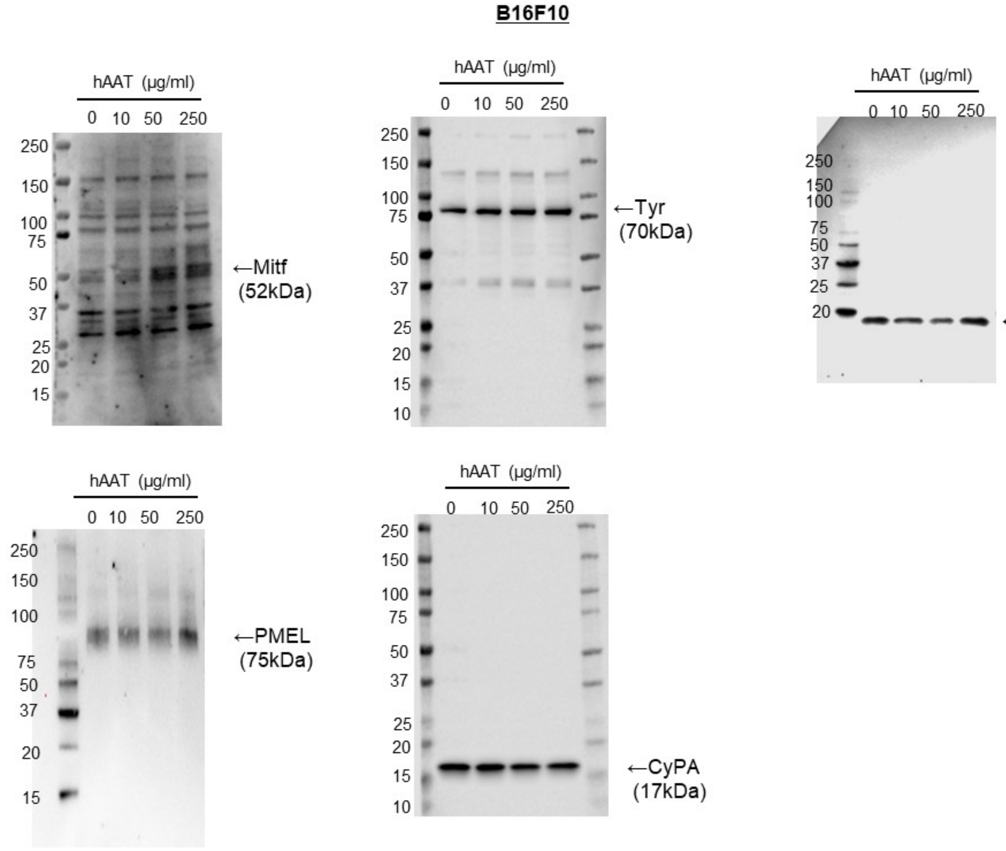

**Figure S5**. Original images of Figure 7A.

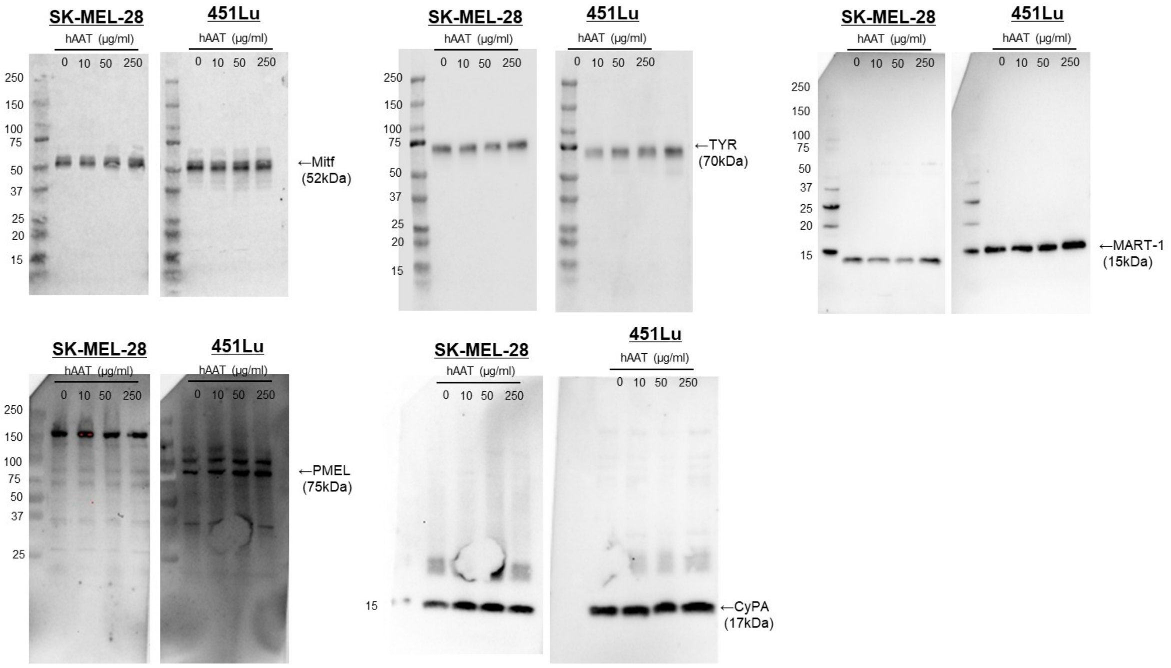

Supplement: Supplementary file 1 [file biomolecules-16-00122-s001.zip › biomolecules-4021962-supplementary.pdf]
